# Novel evaluation scale for QOL (QOL-ACD-BP) in preoperative chemotherapy for breast cancer

**DOI:** 10.1007/s00432-018-2670-0

**Published:** 2018-05-19

**Authors:** Koji Takada, Shinichiro Kashiwagi, Wataru Goto, Yuka Asano, Katsuyuki Takahashi, Tamami Morisaki, Tsutomu Takashima, Shuhei Tomita, Kosei Hirakawa, Masaichi Ohira

**Affiliations:** 1 Department of Surgical Oncology, Osaka City University Graduate School of Medicine, 1-4-3 Asahi-machi, Abeno-ku, Osaka, 545-8585 Japan; 2 Department of Pharmacology, Osaka City University Graduate School of Medicine, 1-4-3 Asahi-machi, Abeno-ku, Osaka, 545-8585 Japan

**Keywords:** Quality of life, Breast cancer, Prognostic marker, Preoperative chemotherapy, QOL-ACD-B

## Abstract

**Purpose:**

Quality-of-life (QOL) has been reported to affect the prognosis of many types of cancer, and several studies used various QOL assessment tools to determine the relationship between QOL and cancer prognosis. In this study, QOL-Questionnaire for Cancer Patients Treated with Anti-Cancer Drugs-the Breast (QOL-ACD-B) was modified to be suitable for preoperative chemotherapy (POC) and was named the QOL-ACD-BP.

**Methods:**

A total of 300 patients were treated with POC after being diagnosed with breast cancer between February 2007 and December 2016 at our institute. We evaluated novel evaluation scale for QOL (QOL-ACD-BP) before and after POC in a retrospective manner.

**Results:**

In the multivariate analysis with overall survival, the high QOL before [*p* = 0.048, hazard ratio (HR) 0.441] or after POC (*p* = 0.030, HR 0.273) was an independent factor.

**Conclusion:**

Our study shows that QOL after POC may also affect prognosis and supported the importance of maintaining QOL in cancer treatment. In patients with breast cancer treated with POC, QOL-ACD-BP, which is a new QOL evaluation index, was found to be a useful tool for predicting the patients’ prognosis.

**Electronic supplementary material:**

The online version of this article (10.1007/s00432-018-2670-0) contains supplementary material, which is available to authorized users.

## Introduction

Recently, psychological and social background as well as therapeutic effects and side effects have become important considerations in selecting cancer treatment (Howell et al. 2015). Quality-of-life (QOL) is the sum of the physical, psychological, and social aspects of an individual. QOL has been reported to affect the prognosis of many types of cancer, and several studies used various QOL assessment tools to determine the relationship between QOL and cancer prognosis (Dancey et al. 1997; Giesinger et al. 2016; Gotay et al. 2008; Kaasa et al. 1989; Maisey et al. 2002). Health-related (HR)-QOL has been set as a secondary endpoint in various clinical trials (Aihara et al. 2014; Howell et al. 2015; Ohsumi et al. 2011; Shiroiwa et al. 2011; Watanabe et al. 2009). Furthermore, HR-QOL has also been considered in the approval of new drugs by the US Food and Drug Administration. Although various QOL measures are available and many of them can adequately evaluate each aspect, some factors overlap and may not be applicable for certain situations. In Japan, the Quality of Life Questionnaire for Cancer Patients Treated with Anti-Cancer Drugs-Breast (QOL-ACD-B) suitable for the Japanese population as a disease-specific scale is often used for patients with breast cancer (Kurihara et al. 1999; Otsuka et al. 2015).

However, because it is a scale for advanced breast cancer, some items were difficult to evaluate in patients administered with preoperative chemotherapy (POC). Therefore, QOL-ACD-B was modified to be suitable for POC and was named the Quality of Life Questionnaire for Cancer Patients Treated with Anti-Cancer Drugs-Breast-Preoperative Chemotherapy (QOL-ACD-BP). In this study, we examined the usefulness of QOL-ACD-BP in patients with breast cancer administered with POC.

## Methods

### Patients’ background

Regarding patients’ characteristics, we targeted the same patients as previously reported (Asano et al. 2016; Goto et al. 2017). A total of 300 patients were treated with POC after being diagnosed with breast cancer between February 2007 and December 2016 at the Osaka City University Hospital. All cases were diagnosed with breast cancer via pathological examination using core-needle biopsy or vacuum-assisted biopsy. In this study, cancer stage and therapeutic effect were evaluated via ultrasonography (US), computed tomography (CT), and bone scintigraphy. Breast cancer was classified into subtypes according to the immunohistochemical evaluation of oestrogen (ER), progesterone (PgR), human epidermal growth factor receptor 2 (HER2), and Ki67.

Breast cancer was categorised into the following immunophenotypes: luminal A (ER^+^ and/or PgR^+^, HER2^−^, Ki67^low^); luminal B (ER^+^ and/or PgR^+^, HER2^+^) (ER^+^ and/or PgR^+^, HER2^−^, Ki67^high^); HER2-enriched breast cancer (HER2BC; ER^−^, PgR^−^, and HER2^+^); and triple-negative breast cancer (TNBC; ER^−^, PgR^−^, and HER2^−^). The cutoff of Ki-67 was set at 14% based on the previous report (Cheang et al. 2009). Luminal A and luminal B types were defined as hormone receptor-positive breast cancer (HRBC) (Goldhirsch et al. 2011). All patients received a standardised protocol of POC consisting of four courses of FEC100 (500 mg/m^2^ fluorouracil, 100 mg/m^2^ epirubicin, and 500 mg/m^2^ cyclophosphamide) every 3 weeks, followed by 12 courses of 80 mg/m^2^ paclitaxel administered weekly. The patients with HER2BC were additionally administered weekly (2 mg/kg) or tri-weekly (6 mg/kg) trastuzumab during paclitaxel treatment (Kawajiri et al. 2012; Mauri et al. 2005; Mieog et al. 2007). All patients received POC in the outpatient department. Therapeutic anti-tumour effects were evaluated according to the Response Evaluation Criteria in Solid Tumors guideline (Eisenhauer et al. 2009). We defined clinical partial response and complete response as ‘responders’ in the objective response rate (ORR), while clinical stable disease and clinical progressive disease were evaluated as ‘non-responders’. Patients underwent surgery (i.e., total mastectomy or breast-conserving surgery) after POC (Kashiwagi et al. 2015). The pathological therapeutic effect of POC was evaluated using resected specimens, and a pathological complete response (pCR) was defined as the complete disappearance of the invasive components of the lesion with or without intraductal components (including that in the lymph nodes) according to the National Surgical Adjuvant Breast and Bowel Project B-18 protocol (Wolmark et al. 2001). All patients with breast-conserving surgery received postoperative radiotherapy to the remnant breast.

The standard postoperative adjuvant treatment was performed according to the breast cancer subtype. Disease-free survival (DFS) was defined as the period between the time of surgery and recurrence. Meanwhile, overall survival (OS) was defined as the time between surgery and death, and follow-up time was defined as the time between surgery and the time that the patient was last examined (death or last visit). Follow-up physical examination was performed every 3 months, US every 6 months, and CT and bone scintigraphy annually. The median follow-up period was 1477 days (range 63–3524 days) from the day of surgery.

### Scoring of QOL-ACD-BP

QOL-ACD was developed by Kurihara and was supported by the Japanese Ministry of Health and Welfare (Kurihara et al. 1999). As it covers all cancers, QOL-ACD-B as a breast cancer disease-specific scale is available (Otsuka et al. 2015) (Supplementary Table 1).

We first evaluated QOL before and after POC using QOL-ACD-B. Before POC, the high QOL group was significantly associated with higher survival rates, both in terms of DFS and OS (date not shown). However, there was no significant difference in DFS or OS due to a QOL difference after POC (date not shown). It was thought that QOL greatly decreased due to symptoms of breast cancer itself, and the progression of breast cancer influenced prognosis. However, we noticed several problems. Because the subjects for QOL-ACD-B are those with advanced breast cancer, any items did not apply to the patients who received POC. On the other hand, few items for side effects and psychological and social aspects were available in QOL-ACD-B. Therefore, we modified a new QOL score to be appropriate for patients administered with POC and named it QOL-ACD-BP in this study. Briefly, QOL-ACD-BP consists of 18 items, each of which is classified into three subscales comprising 6 items, namely, ‘physical aspects’, ‘emotional aspects’, and social aspects’ (Table 1). Other points are the same as QOL-ACD-B, and each item is scored from 1 to 5, with 1 being the worst and 5 being the best. The score of the whole QOL and each subscale was the average of the items that could be evaluated.

**Table 1:** Quality of life questionnaire for cancer patients treated with anti-cancer drugs-Breast-preoperative chemotherapy (QOL-ACD-BP)

*Physical aspects*	
1.	Did you care about pain or numbness caused by disease or treatment?
2.	Did you care about swelling caused by disease or treatment?
3.	Did you care about the skin symptoms (redness, swelling, hotness, itching, hair loss, etc.) caused by disease or treatment?
4.	Did you care about the dietary symptoms (food amount decreased, taste changed, body weight changed, etc.)?
5.	Did you feel disturbing, tired, or insomnia?
6.	Did you have any other concerns in your body (headache, breathlessness, palpitations, eye irritation, etc)?
*Emotional aspects*	
7.	Were you satisfied with the explanation from medical staff (your doctor, nurse, pharmacist, etc.) about the medical condition and treatment?
8.	Do you mind the progress of the current disease and the effect of treatment?
9.	Do you feel uneasy about side effects, future complications, and body changes?
10.	Have you fully accepted your disease?
11.	Have you tried to face up to the disease positively?
12.	Do you feel uneasy about future (treatment period, whether it will be cured or not, whether it comes to life or not)?
*Social aspects*	
13.	Do you feel hesitant to dressing or hesitation for being naked in public in hot springs?
14.	Do you feel uneasy about balancing housework, work and treatment?
15.	Do you feel uneasy about cost?
16.	Are you satisfied with sex life?
17	Are you worried that your family will get the same disease?
18.	Do you feel a sense of alienation, loneliness, and discrimination from the surroundings?

### Evaluation of QOL-ACD-BP

**Fig. 1:**
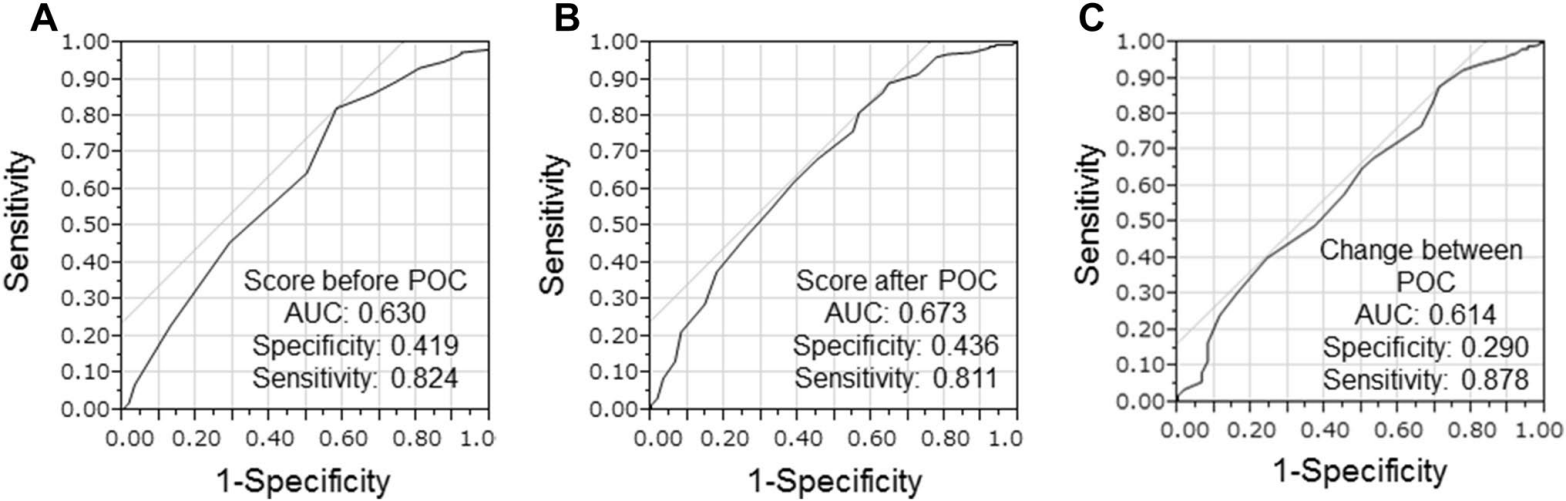
Receiver operating characteristic (ROC) analysis. The median QOL before POC was 4.357 (range 3.071–4.714), and the cut-off value was 4.286 (AUC 0.630, *p* = 0.002, 95% CI 0.549–0.710, sensitivity = 82.4%, specificity = 41.9%) (**a**). Similarly, the median QOL after POC was 3.964 (range 2.214–4.643), and the cut-off value was 3.714 (AUC 0.673, *p* < 0.001, 95% CI 0.595–0.751, sensitivity = 81.1%, specificity = 43.6%) (**b**). Finally, the median change in QOL before and after POC was 0.643 (range − 1.143–1.500), and the cut-off value was 0.214 (AUC 0.614, *p* = 0.006, 95% CI 0.534–0.693, sensitivity = 87.8%, specificity = 29.0%) (**c**)

QOL before POC was retrospectively evaluated using QOL-ACD-BP. Patients receiving chemotherapy, not only for breast cancer but for various cancers, had responded to some questionnaires of medical persons such as nurses and pharmacists. First, in evaluating each item of QOL-ACD-BP, the same items as the other questionnaires were used as they were. Regarding the evaluation of items that did not have the same question in the other questionnaires, if detailed records of remarks on the items remained, they were evaluated using the records. Items that were difficult to evaluate were treated as no answer. Likewise, QOL at preoperative after POC was also evaluated. The change in QOL before and after POC was also calculated. The cut-off value of each QOL was determined using receiver operating characteristic curve analysis to stratify patients at high risk of malignancy-related recurrences. The median QOL before POC was 4.357 (range 3.071–4.714), and the cut-off value was 4.286 [AUC 0.630, *p* = 0.002, 95% confidence interval (CI) 0.549–0.710, sensitivity = 82.4%, specificity = 41.9%] (Fig. 1a). Similarly, the median QOL after POC was 3.964 (range 2.214–4.643), and the cut-off value was 3.714 (AUC 0.673, *p* < 0.001, 95% CI 0.595–0.751, sensitivity = 81.1%, specificity = 43.6%) (Fig. 1b). Finally, the median change in QOL before and after POC was 0.643 (range − 1.143 to 1.500), and the cut-off value was 0.214 (AUC 0.614, *p* = 0.006, 95% CI 0.534–0.693, sensitivity = 87.8%, specificity = 29.0%) (Fig. 1c). Prognosis prediction and factor analysis were performed therefrom.

### Ethics

This study was carried out at the Osaka City University Graduate School of Medicine, Osaka, Japan, according to the REporting recommendations for Tumor MARKer prognostic studies (REMARK) guidelines and a retrospectively written research, pathological evaluation, and statistical plan (McShane et al. 2005). This study was performed in accordance with the provisions of the Declaration of Helsinki (64th WMA General Assembly, Fortaleza, Brazil, October 2013). Written informed consent was obtained from all patients. The Osaka City University Ethics Committee approved the study protocol (Approval number: 926).

### Statistical analysis

All statistical analyses were conducted using the JMP software package (SAS, Tokyo, Japan). The relationship between each factor was examined using chi-squared test. The Kaplan–Meier method and the log-rank test were used for comparison between DFS and OS. The hazard ratios and 95% CIs were calculated using the Cox proportional hazards model. Univariate and multivariate analyses were performed using the Cox regression model. A *p* value of < 0.05 was considered to indicate statistical significance.

## Results

### Correlations between clinicopathological features and QOL-ACD-BP

The clinicopathological features of the 300 patients were the same as that previously reported. The details are listed in Table 2. The median age at surgery was 55 years (range 27–90 years), and the median tumour diameter was 29.0 mm (range 10.2–98.1 mm). Thirty-eight (12.7%) patients had skin infiltration, and 230 (70.0%) were diagnosed with lymph node metastasis at the time of breast cancer diagnosis. A total of 149 (49.7%), 57 (19.0%), and 94 (31.3%) patients were diagnosed with HRBC, HERBC, and TNBC, respectively. The rate of responders for POC from the study cohort was 89.3%, with 99 patients (33.0%) achieving pCR. After surgery, 62 (20.7%) patients had recurrence, and 30 (10.0%) died from breast cancer. Based on the cut-off value described in the ‘Methods’ section, 185 (61.7%) were classified as the high QOL group and 115 (38.3%) as the low QOL group before POC. Similarly, after POC, 228 (76.0%) patients were classified as the high QOL group and 72 (24.0%) as the low QOL group. Finally, in terms of the change in QOL score before and after POC, 47 (15.7%) patients were classified into the low group, that is, the group in which the QOL was significantly decreased by POC. Hereafter, it is called the ‘lower change group’. Meanwhile, 253 (84.3%) patients were classified into the high group, that is, the group which POC did not significantly decrease the QOL, and this group is called the ‘higher change group’.

**Table 2:** Clinicopathological features of 300 patients who were treated with preoperative chemotherapy

Parameters (*n* = 300)	Number of patients (%)
Age (years old)	
≤ 35/36–55/56–70/71 ≤	36 (12.0%)/120 (40.0%)/127 (42.3%)/17 (5.7%)
Tumor size (mm)	
≤ 20/20 <, ≤ 50/50 ≤	35 (11.7%)/ 224 (74.7%)/ 41 (13.7%)
Skin infiltration	
Negative/positive	262 (87.3%)/ 38 (12.7%)
Lymph node metastasis	
N0/N1/N2/N3	90 (30.0%)/116 (38.6%)/65 (21.7%)/29 (9.7%)
Estrogen receptor	
Negative/positive	155 (51.7%)/145 (48.3%)
Progesterone receptor	
Negative/positive	200 (66.7%)/100 (33.3%)
HER2	
Negative/positive	212 (70.7%)/88 (29.3%)
Ki67	
Low/high	96 (32.0%)/204 (68.0%)
Intrinsic subtype	
HRBC/HER2BC/TNBC	149 (49.7%)/57 (19.0%)/94 (31.3%)
ORR	
Non-responders/responders	32 (10.7%)/268 (89.3%)
pCR	
Negative/positive	201 (67.0%)/99 (33.0%)
Reccurence	
Negative/positive	238 (79.3%)/62 (20.7%)
Death from breast cancer	
Negative/positive	270 (90.0%)/30 (10.0%)
QOL-ACD-BP before neoadjuvant chemotherapy	
Low/high	115 (38.3%)/185 (61.7%)
Change of QOL from before POC to after POC	
The lower change/the higher change	47 (15.7%)/253 (84.3%)
QOL-ACD-BP after neoadjuvant chemotherapy	
Low/high	72 (24.0%)/228 (76.0%)

*HER* human epidermal growth factor receptor, *HRBC* hormone receptor-positive breast cancer (ER^+^ and/or PgR+), *HER2BC* human epidermal growth factor receptor 2-enriched breast cancer (ER^−^, PgR^−^, and HER2+), *TNBC* triple negative breast cancer (ER^−^, PgR^−^, and HER2^−^), *ORR* objective response rate, *pCR* pathological complete response, *QOL-ACD-BP* Quality of Life Questionnaire for Cancer Patients Treated with Anti-Cancer Drugs-Breast-Preoperative Chemotherapy

Correlations between the clinicopathological feature and each QOL group are listed in Table 3. Before POC, the tumor size was larger in the low QOL group than that in the high QOL group (*p* < 0.001). Further, in the low QOL group, the rate of skin infiltration and PgR positivity was higher than that in the high QOL group (*p* < 0.001, *p* = 0.029, respectively). After POC, the tumour size was significantly larger and the rate of ‘responder’ was lower in the low QOL group than the high QOL group (*p* = 0.042, *p* = 0.020, respectively). In the ‘higher change group’, the rate of ER positivity was significantly higher compared to the ‘lower change group’ (*p* = 0.014). Moreover, in HRBC, the decrease of QOL by POC was significantly small, while QOL in the TNBC group was significantly decreased (*p* = 0.008, *p* = 0.001, respectively). It also correlated with ORR, and QOL significantly decreased in the non-responders of ORR (*p* = 0.040). No correlation between QOL before and after POC was noted (*p* = 0.319). However, QOL significantly decreased in the high QOL before POC, and the number of patients in the ‘lower change group’ being classified into the low QOL group after POC was higher (*p* < 0.001, *p* < 0.001, respectively).

**Table 3:** Correlations between clinicopathological features and QOL-ACD-BP before and after preoperative chemotherapy

Parameters		QOL-ACD-B before POC		*p* value	Change of QOL		*p* value	QOL-ACD-B after POC		*p* value
		High (*n* = 185)	Low (*n* = 115)		Higher (*n* = 253)	Lower (*n* = 47)		High (*n* = 228)	Low (*n* = 72)	
Age at operation (years)										
≤ 55 > 55		93 (50.3%) 92 (49.7%)	63 (54.7%) 52 (45.3%)	0.449	137 (54.2%) 116 (45.8%)	19 (40.4%) 28 (59.6%)	0.084	123 (53.9%) 105 (46.1%)	33 (45.8%) 39 (54.2%)	0.231
Tumor size (mm)										
≤ 50 > 50		174 (94.1%) 11 (6.0%)	85 (73.9%) 30 (26.1%)	< 0.001	219 (86.6%) 34 (13.4%)	40 (85.1%) 7 (14.9%)	0.791	202 (88.6%) 26 (11.4%)	57 (79.2%) 15 (20.8%)	0.042
Skin infiltration										
Negative Positive		178 (96.2%) 7 (3.8%)	84 (73.0%) 31 (27.0%)	< 0.001	220 (87.0%) 33 (13.0%)	42 (89.4%) 5 (10.6%)	0.650	203 (89.0%) 25 (11.0%)	59 (81.9%) 13 (18.1%)	0.116
Lymph node status										
Negative Positive		62 (33.5%) 123 (66.5%)	28 (24.3%) 87 (75.7%)	0.093	73 (28.9%) 180 (71.1%)	17 (36.2%) 30 (63.8%)	0.316	65 (28.5%) 163 (71.5%)	25 (34.7%) 47 (65.3%)	0.318
Estrogen receptor										
Negative Positive		99 (53.5%) 86 (46.5%)	56 (48.7%) 59 (51.3%)	0.419	123 (48.6%) 130 (51.4%)	32 (68.1%) 15 (31.9%)	0.014	112 (49.1%) 116 (50.9%)	43 (59.7%) 29 (40.3%)	0.117
Progesterone receptor										
Negative Positive		132 (71.4%) 53 (28.6%)	68 (59.1%) 47 (40.9%)	0.029	163 (64.4%) 90 (35.6%)	37 (78.7%) 10 (21.3%)	0.057	147 (64.5%) 81 (35.5%)	53 (73.6%) 19 (26.4%)	0.153
HER2										
Negative Positive		131 (70.8%) 54 (29.2%)	81 (70.4%) 34 (29.6%)	0.945	176 (69.6%) 77 (30.4%)	36 (76.6%) 11 (23.4%)	0.333	160 (70.2%) 68 (29.8%)	52 (72.2%) 20 (27.8%)	0.741
Ki67										
Low High		61 (33.0%) 124 (67.0%)	35 (30.4%) 80 (69.6%)	0.648	80 (31.6%) 173 (68.4%)	16 (34.0%) 31 (66.0%)	0.745	76 (33.3%) 152 (66.7%)	20 (27.8%) 52 (72.2%)	0.380
Intrinsic subtype HRBC										
No Yes		96 (51.9%) 89 (48.1%)	55 (47.8%) 60 (52.2%)	0.495	119 (47.0%) 134 (53.0%)	32 (68.1%) 15 (31.9%)	0.008	109 (47.8%) 119 (52.2%)	42 (58.3%) 30 (41.7%)	0.120
Intrinsic subtype HER2BC										
No Yes		149 (80.5%) 36 (19.5%)	94 (81.7%) 21 (18.3%)	0.798	204 (80.6%) 49 (19.4%)	39 (83.0%) 8 (17.0%)	0.708	185 (81.1%) 43 (18.9%)	58 (80.6%) 14 (19.4%)	0.913
Intrinsic subtype TNBC										
No Yes		125 (67.6%) 60 (32.4%)	81 (70.4%) 34 (29.6%)	0.604	183 (72.3%) 70 (27.7%)	23 (48.9%) 24 (51.1%)	0.001	162 (71.1%) 66 (28.9%)	44 (61.1%) 28 (38.9%)	0.114
ORR										
Non-responders Responders		18 (9.7%) 167 (90.3%)	14 (12.2%) 101 (87.8%)	0.507	23 (9.1%) 230 (90.9%)	9 (19.1%) 38 (80.9%)	0.040	19 (8.3%) 209 (91.7%)	13 (18.1%) 59 (81.9%)	0.020
Pathological response										
Non-pCR pCR		117 (63.2%) 68 (36.8%)	84 (73.0%) 31 (27.0%)	0.080	171 (67.6%) 82 (32.4%)	30 (63.8%) 17 (36.2%)	0.616	149 (65.4%) 79 (34.6%)	52 (72.2%) 20 (27.8%)	0.281
QOL before POC										
Low High		– –	– –		108 (42.7%) 145 (57.3%)	7 (14.9%) 40 (85.1%)	< 0.001	91 (39.9%) 137 (60.1%)	24 (33.3%) 48 (66.7%)	0.319
Change of QOL										
The lower change The higher change		40 (21.6%) 145 (78.4%)	7 (6.1%) 108 (93.9%)	< 0.001	– –	– –		4 (1.8%) 224 (98.2%)	43 (59.7%) 29 (40.3%)	< 0.001
QOL after POC										
Low High		48 (25.9%) 137 (74.1%)	24 (20.9%) 91 (79.1%)	0.319	29 (11.5%) 224 (88.5%)	43 (91.5%) 4 (8.5%)	< 0.001	– –	– –	

*QOL-ACD-BP* Quality of Life Questionnaire for Cancer Patients Treated with Anti-Cancer Drugs-Breast-Preoperative Chemotherapy, *POC*: preoperative chemotherapy, *HER* human epidermal growth factor receptor, *HRBC* hormone receptor-positive breast cancer (ER^+^ and/or PgR+), *HER2BC* human epidermal growth factor receptor 2-enriched breast cancer (ER^−^, PgR^−^, and HER2+), *TNBC* triple negative breast cancer (ER^−^, PgR^−^, and HER2^−^), *ORR* objective response rate, *pCR* pathological complete response

### Correlation between each QOL and prognosis

**Fig. 2:**
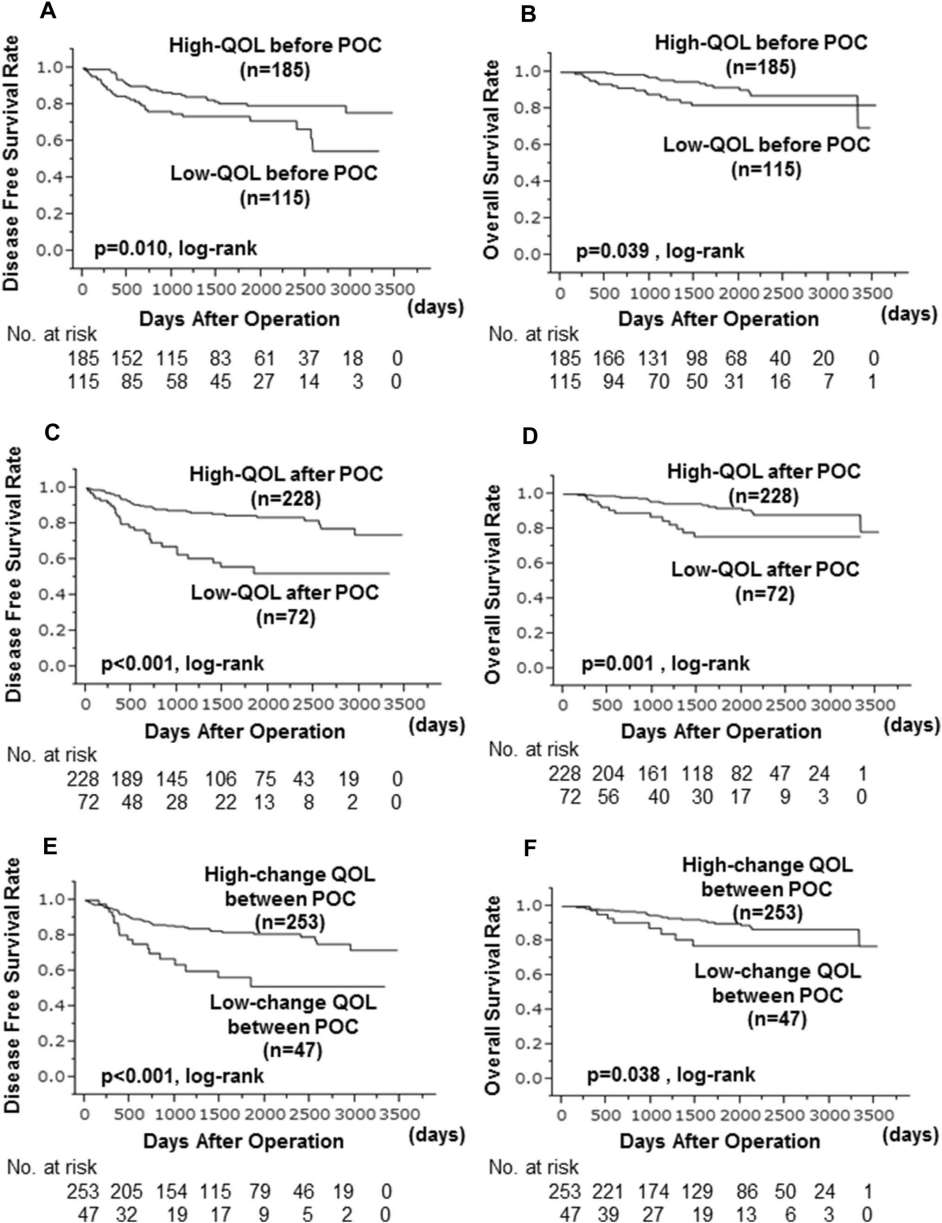
In comparison of disease free survival (DFS) and overall survival (OS) between the high QOL group and the low QOL group before preoperative chemotherapy (POC) the high QOL group was significantly associated with higher survival rates, both in terms of DFS (*p* = 0.010, log-lank) (**a**) and OS (*p* = 0.039, log-lank) (**b**). After POC the high QOL group was significantly associated with higher survival rates, both in terms of DFS (*p* < 0.001, log-lank) (**c**) and OS (*p* = 0.001, log-lank) (**d**). The higher change group and the lower change group of QOL before and after POC the higher QOL group was significantly associated with higher survival rates, both in terms of DFS (*p* < 0.001, log-lank) (**e**) and OS (*p* = 0.038, log-lank) (**f**)

Before POC, the high QOL group was significantly associated with higher survival rates, both in terms of DFS (*p* = 0.010, log-lank) and OS (*p* = 0.039, log-lank) (Fig. 2a, b). Despite the lack of correlation between QOL before and after POC, the high QOL group was significantly associated with higher survival rates even after POC (DFS: *p* < 0.001, log-rank. OS: *p* = 0.001, log-rank) (Fig. 2c, d). Furthermore, the ‘higher change group’ was significantly associated with higher survival rates than the ‘lower change group’ (DFS: *p* < 0.001, log-rank. OS: *p* = 0.038, log-rank) (Fig. 2e, f). In the univariate analysis with DFS, the high QOL group either before or after POC and the ‘higher change group’ were found to significantly contribute to a longer DFS (before POC: *p* = 0.012, HR 0.522; after POC: *p* < 0.001, HR 0.941; change of QOL: *p* = 0.001, HR 0.380). However, in the multivariate analysis, lymph node status (*p* = 0.003, HR 2.816) and response to NAC (*p* < 0.001, HR 0.275) were independent factors, whereas none of the QOL was an independent factor (Table 4). On the other hand, in the univariate analysis of OS, the high QOL either before or after POC was found to significantly contribute to a longer OS (before POC: *p* = 0.045, HR 0.478; after POC: *p* = 0.004, HR 0.321). High QOL before or after POC was also an independent factor in the multivariate analysis (before POC: *p* = 0.048, HR 0.441; after POC: *p* = 0.030, HR 0.273) (Table 5).

**Table 4:** Univariate and multivariate analysis with respect to DFS in 300 cases who were treated with preoperative chemotherapy

Parameters	Univarite analysis			Multivariate analysis		
	Hazard ratio	95% CI	*p* value	Hazard ratio	95% CI	*p* value
Age at operation (years)						
≤ 55	0.693	0.414–1.143	0.151			
> 55						
Tumor size (mm)						
≤ 50	2.882	1.602–4.948	0.001	1.730	0.867–3.299	0.117
> 50						
Skin infiltration						
Negative	2.034	1.056–3.646	0.035	1.162	0.530–2.425	0.700
Positive						
Lymph node status						
Negative	2.426	1.259–5.268	0.007	2.816	1.398–6.315	0.003
Positive						
Estrogen receptor						
Negative	0.751	0.451–1.238	0.262			
Positive						
Progesterone receptor						
Negative	0.928	0.539–1.553	0.781			
Positive						
HER2						
Negative	0.586	0.298–1.063	0.080			
Positive						
Ki67						
Low	0.944	0.563–1.629	0.830			
High						
Intrinsic subtype HRBC						
No	0.757	0.456–1.27	0.274			
Yes						
Intrinsic subtype HER2BC						
No	0.836	0.400–1.574	0.597			
Yes						
Intrinsic subtype TNBC						
No	1.532	0.907–2.539	0.109			
Yes						
ORR						
Non-responders	0.265	0.151–0.492	< 0.001	0.275	0.146–0.540	< 0.001
Responders						
Pathological response						
Non-pCR	0.438	0.222–0.795	0.006	0.578	0.284–1.099	0.097
pCR						
QOL-ACD-BP before POC						
Low	0.522	0.316–0.863	0.012	0.571	0.319–1.036	0.065
High						
Change of QOL between POC						
Lower change	0.380	0.223–0.675	0.001	0.557	0.243–1.222	0.146
Higher change						
QOL-ACD-BP after POC						
Low	0.941	0.923–0.962	< 0.001	0.469	0.234–1.011	0.053
High						

*DFS* disease-free survival, *POC* preoperative chemotherapy, *CI* confidence intervals, *HER* human epidermal growth factor receptor, *HRBC* hormone receptor-positive breast cancer (ER + and/or PgR+), *HER2BC* human epidermal growth factor receptor 2-enriched breast cancer (ER^−^, PgR^−^, and HER2+), *TNBC* triple negative breast cancer (ER^−^, PgR^−^, and HER2^−^), *ORR* objective response rate, *pCR* pathological complete response

**Table 5:** Univariate and multivariate analysis with respect to OS in 300 cases who were treated with preoperative chemotherapy

Parameters	Univarite analysis			Multivariate analysis		
	Hazard ratio	95% CI	*p* value	Hazard ratio	95% CI	*p* value
Age at operation (years)						
≤ 55	0.664	0.311–1.367	0.268			
> 55						
Tumor size (mm)						
≤ 50	2.563	1.070–5.537	0.036	1.318	0.484–3.341	0.576
> 50						
Skin infiltration						
Negative	2.233	0.883–4.969	0.086			
Positive						
Lymph node status						
Negative	3.299	1.164–13.822	0.022	3.490	1.093–15.795	0.034
Positive						
Estrogen receptor						
Negative	0.477	0.213–1.000	0.050			
Positive						
Progesterone receptor						
Negative	0.881	0.394–1.843	0.742			
Positive						
HER2						
Negative	0.29	0.069–0.822	0.017	0.472	0.105–1.566	0.233
Positive						
Ki67						
Low	1.430	0.662–3.427	0.374			
High						
Intrinsic subtype HRBC						
No	0.513	0.233–1.071	0.076			
Yes						
Intrinsic subtype HER2BC						
No	0.498	0.119–1.417	0.210			
Yes						
Intrinsic subtype TNBC						
No	2.850	1.372–6.028	0.005	2.776	1.174–6.871	0.020
Yes						
ORR						
Non-responders	0.227	0.103–0.548	0.002	0.199	0.080–0.522	0.002
Responders						
Pathological response						
Non-pCR	0.376	0.127–0.905	0.028	0.441	0.142–1.146	0.095
pCR						
QOL-ACD-BP before POC						
Low	0.478	0.230–0.982	0.045	0.441	0.196–0.992	0.048
High						
Change of QOL between POC						
Lower change	0.432	0.199–1.039	0.060	1.225	0.345–4.078	0.744
Higher change						
QOL-ACD-B after POC						
Low	0.321	0.154–0.678	0.004	0.273	0.098–0.872	0.030
High						

*OS* overall survival, *POC* preoperative chemotherapy, *CI* confidence intervals, *HER* human epidermal growth factor receptor, *HRBC* hormone receptor-positive breast cancer (ER^+^ and/or PgR+), *HER2BC* human epidermal growth factor receptor 2-enriched breast cancer (ER^−^, PgR^−^, and HER2+), *TNBC* triple negative breast cancer (ER^−^, PgR^−^, and HER2^−^), *ORR* objective response rate, *pCR* pathological complete response

## Discussion

Recently, maintaining QOL in cancer treatment has become important, and QOL is often evaluated as a secondary endpoint in the clinical research of various treatments. QOL has been evaluated using various assessment tools such as the European Organization for Research and Treatment of Cancer Quality of Life Questionnaire-Core 30, Functional Assessment of Cancer Therapy, and Cancer Rehabilitation Evaluation System (CARES) (Aaronson et al. 1993; Cella et al. 1993; Schouten et al. 2016, 2017). Items are comprehensively set so that they can be evaluated according to any carcinoma. As such, questions are diversified, and some items that are not applicable depending on the carcinoma or degree of progression to be investigated are included. In the case of patients with breast cancer, distress and anxiety due to the malignancy are inevitable. A study reported that no significant difference was observed in 63% of clinical trials that used QOL as a secondary endpoint, and the importance of establishing a clear hypotheses and setting subjective items to be investigated before QOL researched has been established (Bottomley and Therasse 2002). Because we measured QOL before and after POC, we determined that QOL-ACD-B is not suitable and changed part of it to be a breast cancer-specific QOL scale in Japan (Kurihara et al. 1999; Otsuka et al. 2015). Herein, we established a new QOL scale and confirmed its practicality.

Before POC, the frequency of skin infiltration and lymph node metastasis was higher (but insignificant) and the tumour size was larger in the low QOL group. This means that QOL before POC is influenced by the degree of disease progression at diagnosis. In advanced breast cancer, QOL decreased because of physical symptoms. Therefore, in the low QOL group, DFS and OS are significantly shorter. Early stage cancer without tumour-related symptoms has been reported to generally have less influence on prognosis (Gotay et al. 2008), and this supports the results of the current study. The appearance of symptoms is hypothesised to decreased that the overall QOL.

The side effect of POC substantially reduces QOL. However, in patients with tumour-related symptoms, their symptoms disappear due to POC; thus, their decline in QOL is limited. Furthermore, the QOL of patients who can be treated with POC is not significantly impaired, and the difference between the maximum and minimum before POC is small. As such, the QOL of patients in the high QOL group before POC significantly decreased and the change of QOL was correlated with QOL after POC.

QOL after POC correlated with ORR of POC. This result can be attributed to the disappearance of tumour-related symptoms via POC and the improvement of the emotional aspect due to the effect of POC. QOL after POC correlating with tumour size suggests that the larger the primary tumour, the better the treatment effect perceived by the patient and improvement in QOL. The correlation between change of QOL and ORR supports this theory. Correlations with QOL, particularly change of QOL, and hormone receptors were particularly observed. The options for drug therapy vary significantly between HRBC and TNBC; thus, the difference in change of QOL will vary according to the choice of treatment. However, only one regimen of anticancer drugs was used in this study, which may be due to the insignificant differences in clinical factors of some subtypes.

QOL and changes in QOL after treatment were reported to be significant predictors of subsequent survival (Coates et al. 1992; Shimozuma et al. 2000). In addition, some studies reported that pain and decreased appetite are poor prognostic factors (Kramer et al. 2000; Lee et al. 2010; Luoma et al. 2003; Spiegel et al. 1989). In this study, no symptom was a prognostic factor. However, overall worsening of the physical symptoms is correlated with the decrease of the overall QOL, and a similar result was reported (Charalambous et al. 2017). Previous studies targeted patients with advanced cancer with distant metastasis, and the correlation between QOL and performance status (PS) has been studied. If QOL and PS decrease, there is little possibility that they will improve thereafter, and treatment options will be reduced, and prognosis will be affected. However, in the current study, because QOL decreased with POC, we believe that the physical symptoms due to the side effects have improved after POC and surgery. However, the temporary decline in QOL affects the patient’s prognosis and options for future treatment.

This research has some limitations such as the limited accuracy of evaluation because the QOL was scored retrospectively using chart records. Prospective studies using a QOL questionnaire are needed to confirm our results.

In conclusion, our study shows that QOL after POC may also affect prognosis and supported the importance of maintaining QOL in cancer treatment. In patients with breast cancer treated with POC, QOL-ACD-BP, which is a new QOL evaluation index, was found to be a useful tool for predicting the patients’ prognosis.

## Electronic supplementary material

Below is the link to the electronic supplementary material.

Supplementary material 1 (DOCX 14 KB)
