# Supplementary material for: Novel evaluation scale for QOL (QOL-ACD-BP) in preoperative chemotherapy for breast cancer
Source: J Cancer Res Clin Oncol. 2018 May 19;144(8):1547–59. doi: 10.1007/s00432-018-2670-0 (PMC6061048; doi:10.1007/s00432-018-2670-0)
Supplement: Supplementary file 1 — Supplementary material 1 (DOCX 14 KB) [file 432_2018_2670_MOESM1_ESM.docx]

**Supplementary Table 1. Quality of Life Questionnaire for Cancer Patients Treated with Anti-Cancer Drugs-Breast (QOL-ACD-B).**

Physical symptoms and pain

1. Did you have pain or numbness in the chest, armpits or arms of the disease side?

2. Did you have swollen arms (swollen) on the disease side?

3. Did the disease side's arms get enough?

4. Were you concerned about the skin symptoms (redness, swelling, hotness, itching, etc.) around the chest on the disease side?

5. Did you have any pain related to disease or treatment?

6. (Please answer this question only for those who underwent surgery) Were you satisfied with the shape of your breasts and surgical scar?

Satisfaction to treatment and coping with disease

7. Were you satisfied with the explanation from your doctor about the medical condition and treatment?

8. Were you satisfied with the hospital facilities and non-doctor staff?

9. Did you accept enough of your disease?

10. Did you think to face the disease?

Side effect to treatment

11. Did you have hair loss?

12. Did you feel tired?

13. Did you suffer from hot flashes and sweating of your body and forehead?

14. Did you suffer from changes in taste (abnormalities)?

Dress, sexual aspect, other

15. Did you feel inconvenienced in clothes, such as you could not wear clothes you want to wear?

16. Did you feel hesitant to being naked in public, such as a hot spring?

17. Are you satisfied with sex life?

18. Were you concerned that your family would get the same disease?
